# UProC: tools for ultra-fast protein domain classification

**DOI:** 10.1093/bioinformatics/btu843

**Published:** 2014-12-23

**Authors:** Peter Meinicke

**Affiliations:** Department of Bioinformatics, Institute for Microbiology and Genetics, University of Göttingen, Germany

## Abstract

**Motivation:** With rapidly increasing volumes of biological sequence data the functional analysis of new sequences in terms of similarities to known protein families challenges classical bioinformatics.

**Results:** The ultrafast protein classification (UProC) toolbox implements a novel algorithm (‘Mosaic Matching’) for large-scale sequence analysis. UProC is by three orders of magnitude faster than profile-based methods and in a metagenome simulation study achieved up to 80% higher sensitivity on unassembled 100 bp reads.

**Availability and implementation:** UProC is available as an open-source software at https://github.com/gobics/uproc. Precompiled databases (Pfam) are linked on the UProC homepage: http://uproc.gobics.de/.

**Contact:** peter@gobics.de.

**Supplementary information:** Supplementary data are available at *Bioinformatics* online.

## 1 Introduction

Next-generation sequencing (NGS) has stimulated the development of many new sequence analysis methods. In particular, metagenomic studies of microbial and viral assemblages require innovative algorithms to analyse the vast amount of anonymous and fragmented sequence data that is obtained from environmental or clinical samples. In a typical bioinformatics pipeline, the assignment of genomic or metagenomic sequences to known protein families is an essential step towards a characterization of the functional repertoire of a particular organism or community. The Pfam (Finn *et al.*, 2010) database of protein families in combination with the HMMER (Eddy, 1998) profile hidden Markov models is widely used for functional annotation of genomic and metagenomic sequences. Before the advent of HMMER 3.0, protein domain detection on large sequence collections had been computationally expensive, and several prefiltering methods (Beckstette *et al.*, 2009; Lingner and Meinicke, 2008; Sun and Buhler, 2007) were suggested to speed up the analysis. Although such a prefilter approach has been included in HMMER 3.0, the processing of large metagenomic sequence files generated by NGS technologies can still be demanding.

In metagenomics and other NGS applications, not only the amount of sequence but also the limited length of sequencing reads can be challenging for a functional analysis (Wommack *et al.*, 2008). Now that Illumina sequencing platforms are increasingly used in metagenomics, we are facing huge collections of short reads often not longer than 100 bp. Here, the question arises what quality of the functional assignments can be expected for sequences that merely cover ∼10% of a typical microbial gene. In a recent study on transcriptomic data, Zhang *et al.* (2013) showed that profile hidden Markov models substantially lose sensitivity on short reads.

We have developed a toolbox for Ultrafast Protein Classification (UProC) that is available in terms of an open-source software. Although UProC can in principle be applied to any protein sequence classification problem, the toolbox is predestined for functional analysis of metagenomes. First of all, the classification speed allows researchers to analyse large metagenomic datasets on a desktop computer without the requirement of large computer clusters or special purpose hardware. In addition, it also provides the necessary functionality to select open reading frames (ORFs) from DNA sequences. Besides the computational speed, our results on simulated metagenome data also indicate that UProC can achieve a considerably higher sensitivity on short reads than profile-based methods.

## 2 System and methods

**Fig. 1.**
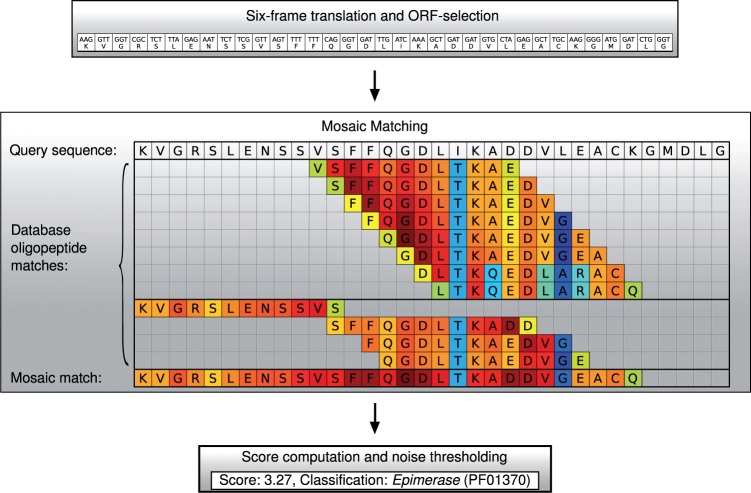
UProC workflow and Mosaic Matching sketch. For DNA input sequences, first all ORFs with at least 60 bp are identified, filtered and translated. The protein sequences then are analysed with the Mosaic Matching algorithm which compares all oligopeptides in the query sequence with oligopeptides from reference sequences in the database. From all matching reference oligopeptides with the same family label a maximum substitution score is computed for each residue and summed up over the whole sequence to provide the total Mosaic Matching score. If this score exceeds a length-dependent noise threshold the protein hit and the corresponding score is written to the output. The substitution scores that result from oligopeptide comparisons using PSSM are indicated by heatmap color (red:high, blue:low). The example shows all matching oligopeptides that contribute to the total score of Pfam family PF01370

The protein sequence classification in UproC is based on a novel algorithm that we refer to as ‘Mosaic Matching’. The algorithm first performs a similarity-based assignment of oligopeptides (‘words’) in the query sequence to protein families in the database. In contrast to *k*-mer based approaches that count the occurrences of short words, in UProC long words (*k* = 18) are scored and classified according to their similarity to ‘neighbouring’ words in the database. The neighbourhood of words is determined by a longest common prefix (LCP) criterion. Finally, all oligopeptides that match the same family are combined to provide a Mosaic Match with a common similarity score for the classification of the entire query sequence. Figure 1 shows an overview of the UProC classification scheme and in the following all elements of the implementation are described in detail.

### 2.1 Database construction

To prepare the oligopeptide database for UProC, all Pfam-A reference sequences from the full-alignment file were analysed. We applied SEG (Wootton and Federhen, 1993) with default parameters for masking of low-complexity regions in all reference sequences. From the remaining regions, we obtained all protein words with a length of 18 amino acids. These reference words were sorted into an array (‘dictionary’). All reference words are stored only once in the database together with their associated protein family labels. Ambiguous words that are associated with multiple protein families are removed from the database. Singleton words in the dictionary whose label is different from their two neighbouring words were removed because there is no evidence that these words provide information for a correct nearest neighbour classification of oligopeptides. For a decreased memory footprint and an accelerated dictionary lookup only the 12-mer suffixes of the oligopeptides are stored explicitly in a 64-bit integer array. For each hexamer prefix, in an additional table we store the dictionary start and end positions of the block of words that share this prefix.

#### Evolutionary alphabet ordering

Before sorting the reference words, we replaced the usual lexicographic order of the alphabet by an ‘evolutionary’ ordering of amino acids to increase the probability that adjacent words in the dictionary belong to the same protein family. Using a 20 × 20 scoring matrix S that had been optimized for discrimination between different protein domain families (Hourai *et al.*, 2004) a distance measure for the elements of the amino acid alphabet was computed from the dissimilarity of column vectors $s_{i}$ of that matrix. For elements *i* and *j* the pairwise distance is

(1) $$d_{ij}=1-\frac{s_{i}^{T}s_{j}}{\left||s_{i}||\;||s_{j}|\right|}.$$

The evolutionary alphabet order was then determined by the shortest cyclic path through all elements yielding an ordering of amino acids according to ATSPGNDEQKRHYWFMLIVC.

### 2.2 Nearest neighbour search

For classification of a new protein sequence each query word is classified and scored according to its nearest neighbours in the database. First, the leading hexamer of the query word is used to identify the block of words in the dictionary sharing the same hexamer prefix. A fast binary search is then performed on the corresponding block to identify an exactly matching reference word, or in the general case, the insertion position of the word in the dictionary together with the two adjacent reference words. The labels of these nearest neighbours associate the query word with the corresponding protein families. Then, all positional substitution scores are computed for the query word with regard to its dictionary neighbours. In this step position-specific scoring matrices (PSSM) are used that have been inferred from supervised learning on all words of the database (see later). The dictionary insertion implies that for the computation of the nearest neighbour oligopeptides in the database, the distance between two words is measured by the word length minus the length of the LCP. Here, the modified lexicographic ordering works as a tie breaking rule to identify the nearest neighbours in the dictionary when the LCP distance is minimal for several reference words. In case of an inexact word match, the labels of both neighbouring reference words are used to label the query word. Also both reference words contribute to the positional scoring using the maximum of the two substitution scores for each position if the labels are identical. The resulting position-specific scores are further used for the successive computation of the Mosaic Matching score. Only the 12 suffix positions of a word are compared and scored because the leading hexamer positions are mostly identical for adjacent words in a large dictionary and thus less informative.

#### Reverse matching

Because the success of a single word match particularly depends on a high conservation of the prefix, we also perform a reverse matching of words. There is a second version of the database dictionary that contains all reference words in reverse order. The reverse matching provides a second chance for a correct word classification if the suffix of the word shows higher conservation than the prefix. The scoring of the reversed words with the second dictionary is computed in the same way as in the normal matching case. The ‘reverse’ scores are combined with the ‘normal’ scores in the final Mosaic Matching step that selects the maximum score for each sequence position. In Figure 1, the four reverse matches are shown in the lower part of the Mosaic Matching sketch.

### 2.3 PSSM learning

A key element of the UProC algorithm is the scoring of protein words with a set of PSSM. The PSSM measure the similarity between neighbouring words and have been computed by means of a supervised machine learning approach. The objective is to optimize the scoring of residues for discrimination of a ‘good’ word match with a correct nearest neighbour classification from a ‘bad’ one that would imply a false classification of that word. In this setup, a positive example is given by two adjacent words in the dictionary with the same protein label. Thus, a positive example represents a correct nearest neighbour match in the database. A negative example is defined by two adjacent words with different class labels *c_i_*,$c_{i+1}$ representing a false match. This gives rise to binary target variables of the learning problem according to

(2) $$y_{i}=\{\begin{array}{cc} +1 & \text{ if}c_{i}=c_{i+1}\\ -1 & \text{else}. \end{array}$$

In turn the binary predictor variables (features) represent the substitutions that have to be applied to transform a word at location *i* to its neighbouring word at location *i* + 1 in the dictionary. Let the indicator vector $x_{i}^{j}$ denote the amino acid at position *j* of a word at location *i*. The substitution at that position with regard to the next word can be represented by a 20×20 binary matrix

(3) $$X_{i}^{j}=x_{i}^{j}x_{i+1}^{jT},x_{i}^{j}\in {\left\{0,1\right\}}^{20}.$$

Among the *k* = 18 residues of a word only the *l* = 12 suffix positions are used for scoring because the hexamer prefix only provides limited information for the discrimination between correct and false matches. Because the sorting of words maximizes the length of the common prefix between adjacent words their leading hexamer positions are mostly identical regardless of the labelling. For training of the *l* PSSM$W_{j}$, a regularized least-squares classifier [see e.g. (Hoff *et al.*, 2008)] was built to discriminate between positive and negative word match examples by minimizing the following prediction error:

(4) $$E(W)=\sum_{i\in I_{\text{train}}}{\left(y_{i}-\sum_{j=1}^{l}\text{tr}(W_{j}^{T}X_{i}^{j})\right)}^{2}+\lambda \sum_{j}\text{tr}(W_{j}^{T}W_{j})$$

where tr indicates the trace operator and $W=[W_{1},\ldots ,W_{l}]$. Note that the sum of traces just realizes a dot product between vectorized and stacked **W** and **X** matrices, respectively. The set of training examples $I_{\text{train}}$ includes all dictionary locations *i* that simultaneously contribute a positive and a negative example, i.e. either $c_{i}=c_{i-1}$ or $c_{i}=c_{i+1}$. All remaining examples are used to validate the regularization parameter *λ* by maximization of the corresponding word match classification rate. Because singleton words in the dictionary (see Section 2.1) provide valuable negative examples, PSSM learning is performed before removing these words from the final database. The computationally efficient least-squares approach to PSSM learning enables to include all words of the dictionary into the optimization which gives rise to a huge training set with several hundred millions of examples. A different strategy would be to choose a random subsample of the dictionary that would allow to use a wider range of machine learning methods such as support vector machines (SVMs) or logistic regression. Furthermore, the reduced training data would also make it possible to apply *n*-fold cross-validation and feature selection techniques. However, this strategy would require to find a suitable trade-off between the size of the training set and the computational complexity of the learning method. Moreover, it has been shown that the regularized least-squares approach can be viewed as a special kind of SVM implementation, also termed proximal or least-squares SVM, which achieved a similar classification performance when compared with state-of-the-art methods on typical benchmark data (Fung and Mangasarian, 2001; Gestel *et al.*, 2004; Zhang and Peng, 2004).

**Fig. 2.**
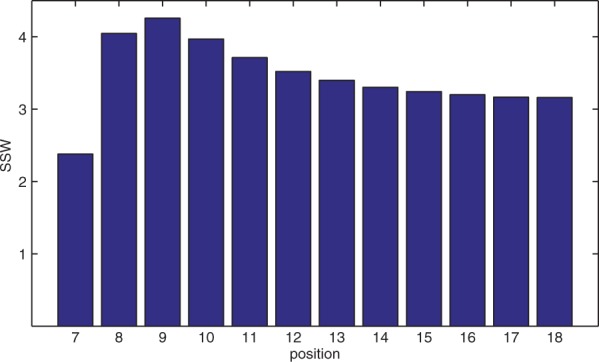
Contributions of different word positions to PSSM in terms of the SSW obtained from regularized least-squares classifier training (see text)

It is important to realize that the PSSM learning scheme makes it possible to automatically balance the impact of different word positions which contribute to the score according to their ability to discriminate between ‘good’ and ‘bad’ matches. Therefore the word length, i.e. the dimensionality of the above feature space, is not a hyperparameter. This role is completely shifted to the regularization parameter *λ*. In principle, this would also enable the use of longer words, which might in fact provide additional information. However, the necessarily resulting decrease of speed and increase of memory were strong arguments against an extension of the word and suffix lengths. In an early stage of the development of the UProC algorithm I also considered the inclusion of the leading hexamer into the scoring. However, in terms of the word classification performance only a marginal improvement could be achieved. The limited benefit of the prefix for scoring is also indicated by the learnt PSSM when looking at the corresponding weights of the linear classifier. Figure 2 shows the relevance of each 12-mer suffix position *j* in terms of the sum of squared weights (SSW) $\text{tr}(W_{j}^{T}W_{j})$ that is contributed by the particular position. It can be seen that word position 9, i.e. the third suffix position, accounts for the maximum SSW. For successive positions the SSW monotonically decreases with a decreasing slope becoming almost flat at the end. The rapid drop in SSW for the first suffix position (word position 7), already signals the loss of relevance for the early word positions.

### 2.4 Mosaic Matching score

By application of the above optimized PSSM to a query word and its nearest neighbour(s) within the dictionary we obtain substitution scores for each position in the word. The position-specific scores from all query words that share a common class label are finally combined to obtain a total similarity score for the whole sequence with respect to the corresponding protein family. In general, the matching words are overlapping so that different position-specific scores are achieved for the same residue. From these scores the maximum is selected and finally the maxima from all residues in the sequence are summed up to obtain the total score for that class (see Fig. 1). The total Mosaic Matching score is computed for each Pfam that achieves at least one nearest neighbour word match.

#### 2.4.1 Score threshold calibration

The total score that is obtained from all words assigned to the same protein family has to exceed a noise threshold to result in a prediction of that family. After the construction of a particular database, a specific threshold is adjusted according to the fraction of false positive predictions (FPs) that is obtained on random protein sequences. The default threshold is chosen to yield a 0.1% false positive rate (FPR) on random data. An alternative 1% threshold can be selected to realize a more sensitive prediction. However, it is not recommended to use the more sensitive threshold without any post-processing of the predictions, as in all cases we observed a rapid increase of false positives with only a modest increase of true positives (TPs).

#### 2.4.2 Random sequences

The random sequences for the false positive (noise) threshold calibration were generated according to independent residues with emission probabilities estimated from the amino acid frequencies in the UniProt database. We determined the score thresholds for a spectrum of six different sequence lengths (32,64,128,256,512,1024 aa) to take into account the increasing FPR for successively longer sequences. The thresholds for intermediate lengths were obtained by interpolation.

### 2.5 UProC tools

The UProC toolbox provides a collection of programs that accomplish different tasks and for an overview of the functionality they are briefly described in the following. uproc-import is required to import the platform independent database files that can for instance be downloaded from the UProC homepage. uproc-dna is the standard program to analyse DNA sequence multifasta files. This one we also used for the evaluation on the test data. Here, it is important to select the appropriate option for the type of sequencing reads. For read lengths below 200 bp the short read mode (option: -s) should be most adequate. In this mode, no prior selection of ORFs is performed and for each sequence all ORFs (>60 bp) are translated and analysed while only the highest scoring significant protein family will be reported. In normal mode (default), the prior ORF selection according to a machine learning model for codon frequencies (Hoff *et al.*, 2008) further increases the detection speed, because only high scoring ORFs are compared with the protein database. In this mode, all significant families are reported which is important for analysis of multidomain proteins. For the protein classification, it is possible to choose between two calibrated sensitivity levels with the option -P *N*. The default is *N* = 3 which is the most specific choice and highly recommended in order to limit the FPR. The output format options allow to compute a histogram of all protein family counts with a small file size (option: -c) or to produce more detailed output (option: -p) that includes the assigned protein families for each input sequence together with the scores and positions in the corresponding ORF. Additional parameters that have to be specified include the directory of the database, the model directory and the input multifasta file. The model directory contains the UProC model file where the most recent version should be downloaded from the UProC homepage. uproc-prot can be used in combination with a prior gene prediction program that has already identified and translated coding regions. Here, the input has to be a multifasta protein sequence file. uproc-makedb can be used to create a UProC database from scratch providing a collection of labelled protein sequences. It is strongly recommended to mask low-complexity regions before, e.g. by using the SEG tool (Wootton and Federhen, 1993). Note that the usually large collection of sequences should well represent the variation within different protein families. uproc-detailed can be useful for diagnostic purposes. For example, the coloured Mosaic Matching graphics of Figure 1 has been generated with this program.

## 3 Results

To evaluate the classification performance of UProC, we implemented a simulation of metagenomic short read data following the approach of Wommack *et al.* (2008) who sampled short fragments from longer sequences to study the read length dependency of similarity-based protein family assignments. We utilized two different types of sequences from which we extracted the simulated short reads. In close correspondence to the approach of Wommack *et al.* (2008), we also used longer metagenomic Sanger reads which were subject to gene prediction and Pfam analysis to obtain a valid labelling of the subsequently extracted fragments. In addition, we also utilized a collection of microbial genomes where we used the available gene annotation to obtain the labelling. This gives rise to two kinds of test datasets with different characteristics: the test data from metagenomic reads contains sequencing errors and the detection of coding regions has to cope with incomplete genes. This provides a realistic read simulation but some errors in the labelling are highly probable. On the other hand, the test data from microbial genomes can be expected to be almost free of sequencing errors and the full-length gene annotation should provide a more reliable labelling. However, the microbial genomes can also be expected to be closer to the known sequences that have been used to create the Pfam database. For that reason, we used an older version of the Pfam database to slightly increase the ‘sequence novelty’ for the Pfam analysis on the test data. Nevertheless, the genome-based test data, in principle, provides an easier trial while the metagenome-based data is more challenging.

### 3.1 Test data

A large amount (≈10 Gbp) of 100 bp short read data was extracted from 895 bacterial genomes of the Human Microbiome Project [HMP; Gevers *et al.* (2012)]. The test data was sampled from all HMP-annotated genes of these microbial reference genomes. In addition, we extracted reads of varying length from metagenomic data of the global ocean sampling (GOS) expedition (Yooseph *et al.*, 2007) and the Guerrero Negro Hypersaline microbial Mat (GNHM) project (Kunin *et al.*, 2008). Although we analysed all reads (≈0.1 Gbp) from the GNHM project, we used an equally sized subset for the GOS data that was randomly selected from Sargasso Sea samples. In both cases, metagenomic reads had been obtained by Sanger sequencing with an average read length of ≈800 bp.

For sequence classification, we chose the Pfam database of protein domain families. To simulate the novelty of metagenomic sequences, we used an older version of the database (vers. 24) that had been built before the HMP genome annotation was available. We compiled six different datasets from distinct HMP body sites with more than 10 associated genomes available in the HMP database (see Table 1). All test sequences were obtained from coding sequences (CDS), i.e. coding regions of the genomes according to the annotation provided by the HMP consortium. The annotation of the microbial reference genomes involves several tools for gene finding and validation, with some variation of the protocols across HMP sequencing centers. The HMP sequencing center-specific annotation protocols and the consensus annotation protocols can be found at the corresponding section of the HMP website (http://hmpdacc.org/tools_protocols/tools_protocols.php).

For the metagenome data from GOS and GNHM samples, we first applied the gene prediction tool FragGeneScan (Rho *et al.*, 2010) to extract coding regions. The tool was used with a Sanger model according to a 0.5% error rate (options: -complete=0 -train=sanger_5). In all cases, for the genome-based as well as for the metagenome-based data, CDS of sufficient length (≥60 bp) were translated and subsequently analysed by the HMMER 3.0 software using hmmscan to identify all Pfam domains. For each protein domain hit the corresponding DNA sequence was labelled as a positive test region, if the domain was above the family specific HMMER gathering threshold and no different domains were detected in the same region with a per-domain *E*-value below 1. We did not use any regions with overlapping Pfam domains for the evaluation and also completely excluded regions with insignificant domain hits, i.e. hits below gathering threshold. Negative labels were exclusively assigned to CDS without any domain hits. To simulate short reads, sequence fragments were randomly extracted from all positive and negative CDS at a 2-fold coverage. Because the focus was on simulation of Illumina short read data, we did not include random variations of sequence length or simulated sequencing errors, which only have a minor impact on the performance when operating with realistic parameters. For a positive test case, it was sufficient that a sequence contained at least 60 bp of a valid Pfam domain hit in one block, and adjacent negative CDS was allowed to add up to the simulated read length in this case. In that way also shorter protein domains that did not cover the full read length could be included in the evaluation. Although for the larger HMP test data we used a read length of 100 bp throughout, for the smaller GOS and GNHM data we generated subsets of short reads simulating different read lengths of 100, 150, 200 and 250 bp. Note that the fraction of reads with a valid Pfam annotation, i.e. the proportion of positive test cases, increases with read length. The final number of generated reads in all HMP, GOS and GNHM subsets together with the corresponding percentage of positive examples is shown in Table 1. All test datasets together with the corresponding result tables (csv) can be obtained from http://uproc.gobics.de/downloads/test_data/.

**Table 1.** Test data subsets from HMP, GOS and GNHM with number of genomes, number of simulated reads and the percentage of reads with annotated Pfam domains, i.e. the fraction of positive test cases.

Source	Subset	No. genomes	No. reads	% Pfams
HMP	Airways	51	2 268 424	66.7
	Blood	42	2 057 852	67.4
	GI tract	363	22 224 068	61.8
	Oral	193	7 749 640	61.0
	Skin	114	5 724 140	66.0
	UG tract	132	4 494 476	64.4
GOS	100 bp	-	742 527	76.6
	150 bp	-	492 466	81.3
	200 bp	-	361 977	84.9
	250 bp	-	279 125	87.7
GNHM	100 bp	-	415 630	68.9
	150 bp	-	272 129	74.6
	200 bp	-	197 806	79.0
	250 bp	-	150 548	82.8

### 3.2 Performance comparison

To compare UProC with state-of-the-art profile methods for protein domain detection we also measured the classification performance of HMMER and RPS-BLAST (Marchler-Bauer *et al.*, 2002) on the same short read test data. RPS-BLAST is based on PSSM and because it is much faster than HMMER 2 it has been used in some early metagenome projects [see e.g. Kunin *et al.* (2008)].

On the test data, we used HMMER3 and RPS-BLAST with an *E*-value cutoff of 0.01. We first applied hmmscan (options: -noali -domE 1 -domtblout) from HMMER 3.0 and rpsblast (options: -evalue 1 -outfmt 6 -num_threads 4) with a high threshold and then filtered the results according to the chosen *E*-value cutoff. In that way, we could also investigate the performance for a varying cutoff on the GOS and GHNM datasets to ensure that the 0.01 threshold provides a good compromise with a sufficient specificity across all read lengths that compares well with the UProC performance (see Supplementary Tables S1 and S2). UProC was used in short read (best hit) mode with the default noise threshold (0.1% FPR). For HMMER and RPS-BLAST, we provided a six-frame translation of DNA sequences and selected all ORFs with at least 60 bp length. As in UProC short read mode, only the most significant protein hit on a read was evaluated. For the HMMER3 and RPS-BLAST models, we used all Pfam 24 profiles and for the UProC oligopeptide database we used all Pfam 24 sequences from full alignments. The performance was measured in terms of prediction sensitivity and specificity on the HMP, GOS and GNHM datasets. The sensitivity or true positive rate (TPR) was estimated from the number of TP, i.e. the annotated protein domains that were actually detected by a particular method and the number of false negatives (FNs), i.e. the annotated protein domains that have not been detected. The specificity was measured by the positive predictive value (PPV) and additionally requires the number of FPs, i.e. reported protein domain hits in sequences without or with differing Pfam annotation. From these counts, we obtain the two performance indices:

$$\text{TPR}=\frac{\text{TP}}{\text{TP+FN}},\text{PPV}=\frac{\text{TP}}{\text{TP+FP}}\text{.}$$

Although we observed a similarly high specificity above 94% for all tools on the test data, UProC consistently outperforms the profile methods in terms of a higher sensitivity on HMP short reads (see Tables 2 and 3). Here, the profile-based methods detect only about half of the annotated domains in the short reads with HMMER (50.2%) being slightly more sensitive than RPS-BLAST (46.6%). In comparison with the profile methods, UProC showed a higher variation of sensitivity on HMP data ranging from 76.4% (Oral) to 88.9% (Blood). Most probably this variation reflects the different degrees of sequence novelty in the body site-specific subsets. Also on 100 bp short reads from GOS and GNHM datasets, UProC outperforms the profile-based methods, but here the differences become much smaller. An increasing read length strongly improves the predictions of the profile-based methods and HMMER becomes the leading method at 250 and 150 bp for GOS and GNHM data, respectively. In particular, the results on the GNHM data suggest that on longer reads profile-based methods can better cope with the high degree of sequence novelty that is encountered with microbial mat communities.

**Table 2.** Sensitivity (TPR) of protein domain detection on simulated short reads with best value in bold face

Source	Subset	HMMER	RPS-BLAST	UProC
HMP	Airways	52.4	48.6	**84.7**
	Blood	52.3	48.5	**88.9**
	GI tract	49.2	46.0	**85.7**
	Oral	49.3	46.0	**76.4**
	Skin	48.6	45.3	**82.2**
	UG tract	49.2	45.5	**83.7**
GOS	100 bp	47.5	44.8	**68.5**
	150 bp	67.4	61.4	**75.1**
	200 bp	77.3	70.6	**78.7**
	250 bp	**83.4**	76.7	80.9
GNHM	100 bp	42.8	39.6	**50.1**
	150 bp	**62.8**	56.7	58.0
	200 bp	**73.4**	66.7	62.5
	250 bp	**80.1**	73.1	65.7

**Table 3.** Specificity (PPV) of protein domain detection on simulated short reads with best value in bold face

Source	Subset	HMMER	RPS-BLAST	UProC
HMP	Airways	97.5	**98.7**	97.5
	Blood	97.9	**98.9**	98.1
	GI tract	97.1	**98.2**	97.2
	Oral	97.2	**98.3**	97.3
	Skin	97.1	**98.0**	97.5
	UG tract	97.6	**98.6**	97.8
GOS	100 bp	98.6	98.1	**98.9**
	150 bp	**98.2**	97.7	98.1
	200 bp	**96.8**	96.2	96.5
	250 bp	**94.7**	94.1	94.3
GNHM	100 bp	97.8	**98.1**	97.5
	150 bp	**97.8**	97.4	97.4
	200 bp	**97.0**	96.3	96.3
	250 bp	**95.7**	95.0	94.7

### 3.3 Runtime comparison

For measuring the computational classification speed of the compared tools, we analysed the largest metagenomic sequence file from the HMP database (SRS017007) which comprises 13 Gbp of DNA short read data. All tools were run with Pfam 24 on the same desktop computer with 32 GB RAM and an Intel 2.3 GHz quad-core CPU enabling multithreaded computation. UProC was able to process the file in 56 min which corresponds to a speed of 4 megabases per second. Because the profile methods are significantly slower we only processed a fraction (1/2000) of the data with HMMER 3.0 using hmmsearch and RPS-BLAST using rpsblast and extrapolated the runtime. The speed for HMMER and RPS-BLAST was 8.2 and 4.1 kilobases per second, which corresponds to an extrapolated runtime of 19 and 38 days, respectively. Using HMMER 3.1b1, we only observed a minor increase of speed reducing the extrapolated runtime by 1 h in comparison with version 3.0. The runtime of UProC gradually increases with the size of the oligopeptide database. Using Pfam 27 which yields a database more than twice as large than the corresponding Pfam 24 database, UProC needs 58 min to analyse the HMP file. However, the required RAM for UProC grows linearly with the database size. Although UProC with Pfam 24 can be run on notebooks with 8 GB RAM, the larger Pfam 27 database requires more than twice the amount. About 10% more RAM is required for database creation than for sequence classification. In comparison with the short classification runtimes, the creation of an oligopeptide database is a more time consuming process that took 6.5 h for the Pfam 24 database. For that reason, we offer precompiled versions of the Pfam 24 and 27 databases for download from the UProC homepage.

## 4 Discussion

The results on simulated short reads encourage the use of UProC for large-scale metagenome analysis. For 100 bp reads the usual speed-accuracy trade-off seems to be obsolete since UProC is both faster and more sensitive. The computationally more expensive profile methods that provide state-of-the-art classification performance on full-length protein sequences might not be optimal for this kind of short read data. This finding agrees well with a recent study on transcriptomic data (Zhang *et al.*, 2013) where HMMER and other profile-based methods showed inherent difficulties in classifying protein domains on short reads from weakly conserved regions. In our case, this is even more remarkable, because the HMMER program was also used for preparing all test examples on the basis of its predictions on full-length sequences. The sensitivity rates clearly show that on 100 bp short reads HMMER does not recognize a considerable fraction of the protein domains that have been predicted in longer sequences before. For increasingly longer reads HMMER successively outperforms UProC in terms of sensitivity, while in terms of speed it remains ∼500 times slower. It is important to note that UProC requires a large protein database that well represents the sequence variation within different families. In particular, smaller databases that are based on full-length protein sequences might require a homology extension before being used with UProC. This shows an important advantage of profile-based methods that can even be used to represent tiny families with just a few sequences. Furthermore, it can also be expected that profile methods can better cope with the increasing overlap between families in full-length databases. In turn, UProC is not restricted to protein families that are well representable by multiple alignments. At the UProC homepage, we offer a precompiled database for a recent version of the KEGG orthologs (Kanehisa and Goto, 2000) which are widely used for metabolic profiling in metagenomics and metatranscriptomics. Beyond functional analysis, UProC can also be used for fast taxonomic profiling of metagenomes forwarding the protein domain hit counts to the Taxy-Pro mixture model toolbox (Klingenberg *et al.*, 2013).

## Supplementary Material

Supplementary Data
