# Supplementary material for: UProC: tools for ultra-fast protein domain classification
Source: Bioinformatics. 2014 Dec 23;31(9):1382–8. doi: 10.1093/bioinformatics/btu843 (PMC4410661; doi:10.1093/bioinformatics/btu843)
Supplement: Supplementary Data [file supp_31_9_1382__index.html]

UProC: tools for ultra-fast protein domain classification — UProC: tools for ultra-fast protein domain classification — UProC: tools for ultra-fast protein domain classification — Supplementary Data 

# UProC: tools for ultra-fast protein domain classification

## Supplementary Data

files

**Files in this Data Supplement:**

- Supplementary Data - pdf file
