# Supplementary material for: UProC: tools for ultra-fast protein domain classification
Source: Bioinformatics. 2014 Dec 23;31(9):1382–8. doi: 10.1093/bioinformatics/btu843 (PMC4410661; doi:10.1093/bioinformatics/btu843)
Supplement: Supplementary Data [file supp_btu843_uproc-supplement.pdf]

Supplementary material on "UProC: tools for ultra-fast protein domain classification"

Peter Meinicke
Bioinformatics Department, University of Göttingen

Supplementary Tables

We investigated the variation of the HMMER3 and RPS-BLAST prediction performance for a varying E-value cuttoff on the GOS and GHNM datasets. In that way we could ensure that the chosen 0.01 threshold on the test data provides a good compromise with a sufficient specificity across all read lengths that compares well with the UProC performance. The corresponding sensitivity in terms of the true positive rate (TPR) and the specificity in terms of the positive predictive value (PPV) are shown in Table 1 and 2 for HMMER and RPS-BLAST, respectively.

source	read length	measure	E-value 10^{-3}	10^{-2}	10^{-1}	1
GOS	100bp	TPR	39.2	47.5	56.8	57.7
		PPV	99.7	98.6	90.4	88.7
	150bp	TPR	60.6	67.4	75.3	76.2
		PPV	99.3	98.2	90.2	88.6
	200bp	TPR	72.1	77.3	83.8	84.7
		PPV	97.9	96.8	89.7	88.3
	250bp	TPR	79.3	83.4	88.9	89.7
		PPV	95.6	94.7	88.7	87.5
GNHM	100bp	TPR	34.4	42.7	52.5	53.6
		PPV	99.5	97.8	87.3	84.9
	150bp	TPR	49.0	55.3	62.7	63.8
		PPV	99.3	97.8	87.3	84.8
	200bp	TPR	67.6	73.4	80.9	82.1
		PPV	98.3	97.0	88.0	85.7
	250bp	TPR	75.1	80.1	86.3	87.4
		PPV	96.9	95.7	88.2	86.3

Table 1: Sensitivity (TPR) and specificity (PPV) of HMMER for different E-value cutoffs on Global Ocean Sampling (GOS) and Guerrero Negro Hypersaline Microbial Mat (GNHM) datasets.

source	read length	measure	E-value 10^{-3}	10^{-2}	10^{-1}	1
GOS	100bp	TPR	37.7	44.8	53.6	74.2
		PPV	99.8	98.9	90.3	59.5
	150bp	TPR	55.0	61.4	70.0	89.7
		PPV	99.2	98.1	89.4	65.5
	200bp	TPR	65.0	70.6	78.6	94.6
		PPV	97.5	96.5	88.2	68.7
	250bp	TPR	71.8	76.7	83.7	96.6
		PPV	95.2	94.3	87.5	72.1
GNHM	100bp	TPR	32.3	39.6	49.4	75.2
		PPV	99.7	98.1	83.8	47.4
	150bp	TPR	49.7	56.7	67.0	91.0
		PPV	99.3	97.4	82.7	54.3
	200bp	TPR	60.4	66.7	76.4	95.2
		PPV	98.1	96.3	83.2	60.9
	250bp	TPR	67.5	73.1	82.0	96.9
		PPV	96.6	95.0	84.0	66.1

Table 2: Sensitivity (TPR) and specificity (PPV) of RPS-BLAST for different E-value cutoffs on Global Ocean Sampling (GOS) and Guerrero Negro Hypersaline Microbial Mat (GNHM) datasets.
